# Autoimmune diseases as pre-existing conditions and sequelae of post COVID-19 condition in a Massachusetts community based observational study of COVID-19 patients

**DOI:** 10.1371/journal.pone.0337848

**Published:** 2025-12-03

**Authors:** Susan R. Sama, Rebecca Gore, Ann Z. Bauer, Lawrence Garber, Richard Rosiello, Meagan Fair, David Kriebel

**Affiliations:** 1 Department of Public Health, University of Massachusetts Lowell, Lowell, Massachusetts, United States of America; 2 Reliant Medical Group, Inc., Worcester, Massachusetts, United States of America; Freelance Medical Research and Writing, UNITED KINGDOM OF GREAT BRITAIN AND NORTHERN IRELAND

## Abstract

Between 10%−26% of COVID patients develop Post COVID condition (PCC). The complex interaction between autoimmunity and SARS-CoV-2 is emerging as an important challenge and an opportunity to improve diagnosis and treatment of immune mediated chronic illnesses. In a retrospective cohort study using electronic health records from a Massachusetts group medical practice, we identified 38,327 patients with a COVID-19 diagnosis and 1,143 with a PCC diagnosis from 1/1/2020 to 6/25/2023. We investigated the hypotheses that auto-immune diseases-1) increase risk of developing PCC; 2) were more likely to develop after COVID-19; and 3) medical utilization would be higher in patients with a PCC diagnosis. We compared COVID-19 patients with and without a PCC diagnosis. We evaluated demographics, PCC symptoms, pre-infection comorbidities, autoimmune diseases pre- and post- SARS-CoV-2 infection, and medical utilization. Females were more likely to have a PCC diagnosis (63%, p = 0.012). High BMI (> 30), pre-infection chronic respiratory disease, and “any post-infection autoimmune disease” were also associated with PCC diagnosis, OR= 1.25, (95% CI: 1.11, 1.41); OR=1.64, (95% CI: 1.45, 1.86), OR=1.57, (95% CI: 1.10, 2.24), respectively. Pre-infection, psoriasis OR=1.41 (95% CI: 1.04, 1.91) and rheumatoid arthritis OR=1.64, (95% CI: 1.00, 2.69) were more likely to be observed in patients with a PCC diagnosis. Post-infection, Sjögren’s syndrome, OR=4.05 (95% CI: 1.94, 8.49) was more likely among PCC diagnosed patients and rheumatoid arthritis OR=3.18 (95% CI: 0.99, 10.46) may also be more prevalent. We observed approximately one more day of medical utilization per month among patients with a PCC diagnosis (p < 0.001). We confirm PCC diagnosis is more prevalent among women, patients with high BMI and chronic respiratory disease. Our findings support emerging evidence that pre-existing autoimmune diseases may increase risk of PCC, SARS-CoV-2 may increase the risk of new onset autoimmune disease, and medical utilization is higher among patients with PCC.

## Introduction

The global impact of the Coronavirus Disease 2019 (COVID-19) pandemic has been unprecedented, with millions of individuals experiencing a range of symptoms during and after the acute phase of infection. Emerging evidence suggests that between 10–26% of individuals continue to experience persistent multisystem symptoms long after their initial recovery, a condition commonly referred to as Post COVID condition (PCC), Long COVID or post-acute sequelae of SARS-CoV-2 infection (PASC) [1–4]. Better characterization of the clinical manifestations of PCC, including potential associations with autoimmune disorders and evaluating medical utilization rates among PCC patients are crucial for effective prevention and healthcare management.

SARS-CoV-2 infection includes a range of typical flu-like symptoms, and the illness can be mild, moderate or severe. Severe cases have been associated with a substantial inflammatory response called a “cytokine storm” with pro-inflammatory cytokines and chemokines that promote pulmonary inflammation [5]. Recent studies have highlighted the multifaceted nature of a wide range of PCC symptoms, which can include fatigue, dyspnea, cognitive impairment, myalgia, persistent loss of smell and taste and postural orthostatic tachycardia syndrome [1,6]. These symptoms can persist for weeks, months or years and can result in disability [6,7]. However, in most cases PCC patients see improvement after 3–6 months [8].

Emerging evidence suggests that SARS-CoV-2 infection and PCC may also be associated with the exacerbation of pre-existing autoimmune conditions or an increased risk of developing autoimmune disorders [9–16]. Some evidence points to an increased risk of developing PCC among patients with pre-existing autoimmune diseases [17,18]. Autoimmune diseases result when the immune system malfunctions and mistakenly attacks healthy cells, tissues and organs. Autoimmune diseases are increasing and are the third leading cause of morbidity, affecting as many as 50 million Americans [19]. Triggering of autoimmune diseases by viral infections has been suggested before [13,20,21]. This potential link between PCC and autoimmune disorders warrants thorough investigation to elucidate underlying mechanisms and to guide clinical management strategies.

The complex interaction between autoimmunity and SARS-CoV-2 infection is emerging as both an important challenge to medical care and an opportunity to improve diagnosis and treatment of other immune-mediated chronic illness. This paper aims to contribute to the existing literature by characterizing a Massachusetts community-based population of COVID-19 patients comparing patients with a PCC diagnosis to those with no PCC diagnosis in terms of both pre-existing conditions and sequelae. By analyzing electronic health records from our population, we seek to investigate: 1) common PCC symptoms; 2) explore potential associations between pre-SARS-CoV-2 infection autoimmune disorders, and post-SARS-CoV-2 infection autoimmune disorders; and 3) examine rates of healthcare utilization among PCC patients. The overarching goal of this research is to inform clinical practice guidelines, improve patient outcomes, and guide public health strategies in managing the long-term consequences of COVID-19.

## Materials and methods

### Study design and data source

As in our previous studies [22,23], we used de-identified electronic health record (EHR) data from a large health care system, Reliant Medical Group (RMG) serving Central Massachusetts to conduct a retrospective cohort study that followed COVID-19 patients. We have prospectively expanded this community-based cohort to include 38,327 patients diagnosed with COVID-19 and 1,143 diagnosed with PCC in central Massachusetts, USA. We complied with REporting of studies Conducted using Observational Routinely-collected Data-RECORD guidelines for reporting this study. (See S2 Appendix RECORD Checklist.)

### Participants and data collection

Study data on all COVID-19 patients were abstracted and de-identified by the RMG Data Analyst on 6/26/2023 for the time period of 1/1/2020–6/25/2023. EHR data including encounter diagnosis codes, medication order data, medical problem notes and demographics (age, gender, body mass index (BMI), smoking status) were extracted for all patients in the study cohort. (See Supplemental Material S2: Description of Study Codes and Algorithms and Supplemental Tables for more detail.)

When there were multiple reports of symptoms, diagnoses, biomarkers or medications, the value immediately preceding the first positive COVID-19 test (or Long-COVID diagnosis for the 1% with an PCC diagnosis but no acute COVID-19 test/diagnosis) was used.

We extracted all COVID-19 patients using 1) CPT codes for SARS-COV-2 reverse transcription polymerase chain reaction, RT-PCR (87635.xx and 87798.189, 87636.01), rapid antigen tests (87426.xx) with positive result, and/or 2) ICD-10 Code U07.1. Among this group of patients, we then used ICD10 Code U09.9 to identify patients with a PCC diagnosis. WHO defines U09.9 as Post COVID-19 Condition [24].

To evaluate the risk of potential misclassification bias, from 10/4/23–11/2/23 RMG project staff conducted a chart review on a randomized subset of patients with a PCC diagnosis (ICD10 U09.9) and no positive test or diagnosis code (ICD10 U07.7) for COVID-19, to confirm PCC diagnosis and determine whether a COVID-19 diagnosis was noted in the EHR. Our chart review confirmed that 100% of these cases had a note indicating a prior COVID-19 diagnosis. Deidentified summary results were provided to the study team.

As in our prior studies [22,23], encounter and diagnostic codes were used to identify patients as having the following co-morbidities: hypertension (HT), diabetes, chronic respiratory disease, arterial disease, congestive heart failure, immunosuppressed conditions (HIV or history of solid organ transplant), chronic kidney disease, chronic liver disease, cancer, and polycystic ovary syndrome (PCOS-among women only). (See Supporting Information- S2 Appendix-Description of Study Codes and Algorithms and Supplemental Tables for specific codes that were utilized for data extraction.)

Pre-infection autoimmune diseases were those first diagnosed prior to the first COVID diagnosis (or if that was missing the first PCC diagnosis). Post-infection autoimmune diseases were those first diagnosed after the first COVID-19 diagnosis (or PCC diagnosis).

The “any autoimmune disease” variable includes any of the following diagnoses prior to COVID-19/ PCC diagnosis: Addison disease, celiac disease, Graves’ disease, Hashimoto thyroiditis, inflammatory bowel disease (Crohn’s diagnosis, ulcerative colitis), multiple sclerosis, myasthenia gravis, pernicious anemia, reactive arthritis, rheumatoid arthritis, Sjögren’s syndrome, systemic lupus erythematosus (lupus), psoriasis, Lyme disease, chronic fatigue syndrome, immunodeficiency following Epstein Barr. (See Supporting Information- S2 Appendix-Description of Study Codes and Algorithms and Supplemental Tables for relevant IDC 10 Codes used for identification.) We assessed potential confounding by COVID vaccination status, by restricting the ”any autoimmune disease” analyses for both pre- and post-infection to only those who had at least one COVID-19 vaccination.

### Statistical analysis

Routine data verification and cleaning were conducted. We summarized continuous data using mean values and standard deviations and categorical data using counts and percentages. Differences in demographics, PCC symptoms and pre-existing comorbidities including select autoimmune diseases and medical utilization rates (encounter days per month) between patients with and without PCC diagnosis were compared using the Chi-square test for categorical variables (gender, symptoms, comorbidities, smoking status) or t-tests and Wilcoxon tests for continuous variables (diastolic and systolic BP and age, and BMI respectively). Logistic regression models then were fit to evaluate the association between PCC diagnosis and these factors both crudely and while controlling for age (continuous) and gender. We defined statistical significance as p value less than 0.05. In logistic regression models 95% confidence intervals are provided.

We explored COVID-19 and PCC diagnosis by patient demographics including age, gender, race (White, Black, Asian, Other, Unknown), ethnicity (characterized as Hispanic, Not Hispanic, Unknown), BMI, and smoking (ever/never). The standard clinical guideline for obesity of 30 was used to define high BMI. Potential confounders: age (continuous), gender, BMI (continuous), and smoking status (ever/never) were also extracted from the EHR for each patient. For PCC symptoms, we recorded those reported more than 30 days past a COVID-19 or Post COVID condition diagnosis.

We also examined medical utilization in relation to PCC diagnosis, overall and stratified by age (<65, ≥ 65) and gender. Medical utilization was counted as one encounter per day after the first COVID-19 diagnosis or if that was not available, after first PCC diagnosis. This outcome represents the mean number of medical encounter-days per month of follow-up time post COVID-19.

Observations with missing data were excluded from any analysis including that variable. All statistical analyses were conducted using SAS 9.4 (SAS Institute Inc., Carey, NC, USA)

### Ethical considerations

All study activities were performed in accordance with relevant guidelines and regulations. Only adults (age 18 and older) were included, and the study was reviewed and approved via expedited review by the UMASS Lowell Institutional Review Board: IRB number: 20–055 with a waiver of informed consent.

## Results

### Source of COVID-19 or Post COVID condition diagnosis

**Fig 1:**
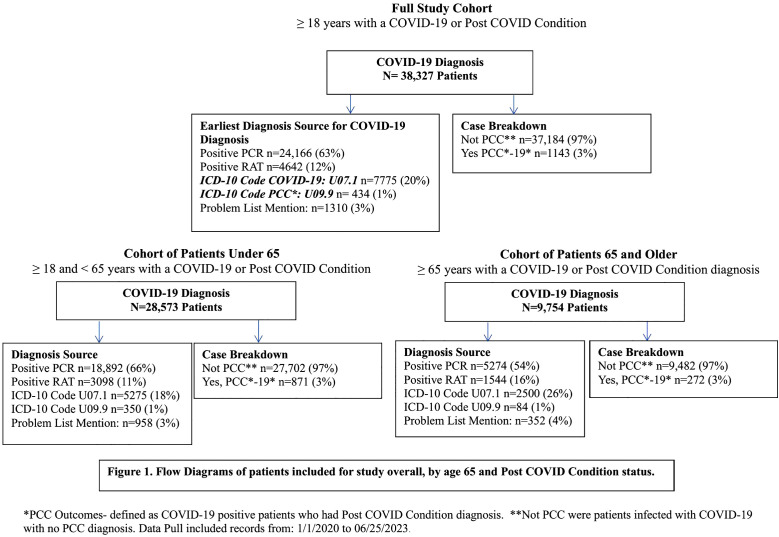
Flow Diagrams of patients included for study overall, by age 65 and Post COVID condition status.

During the time period of 1/1/2020 to 6/25/2023, we identified a total of 38,327 COVID-19 patients, Post COVID condition (n = 1143) and COVID-19 only (n = 37,184) in the central Massachusetts group medical practice. The majority (63.05%, n = 24,166) had a positive reverse transcription polymerase chain reaction (RT-PCR) test, 20% (n = 7775) had a COVID-19 ICD-10 diagnosis code, 12% (n = 4642) had a positive COVID-19 rapid antigen test, approximately 3% (1310) had COVID-19 diagnosis noted in their problem list, and one percent (n = 434) had an ICD-10 diagnosis code for Post COVID condition, and no encounter associated with a specific COVID-19 diagnosis. (Fig 1) The chart review of patients with a PCC diagnosis (ICD10 U09.9) and no positive test or diagnosis code (ICD10 U07.7) for COVID-19 found that in all cases patients had a PCC diagnosis and a prior acute SARS-CoV-2 infection was mentioned in a non-abstracted EHR field, typically in a provider note.

### Demographics

Consistent with other studies, we found that females were more likely to be diagnosed with PCC (63%), as compared to males (p = 0.012) and that high BMI (> 30) was also associated with increased risk of PCC diagnosis (p < 0.001) [25]. (Table 1) We also observed a significantly higher percentage of whites with a PCC diagnosis than expected (p < 0.001). Smoking status (ever versus never) did not appear to be associated with PCC diagnosis. Healthcare utilization was significantly greater by approximately 1 medical-encounter day per month among patients with a PCC diagnosis compared to those COVID-19 patients without a PCC diagnosis (p < 0.0001). PCC diagnosis was more likely to be observed among non-Hispanics and those with unknown ethnicity than Hispanics (p < 0.032). Mean systolic and diastolic blood pressures were within normal range and did not differ between patients with and without a PCC diagnosis.

**Table 1:** Demographics, Baseline Characteristics of Post COVID condition-19*.

Variables	Total COVID-19 Patients	Yes Post COVID condition	No Post COVID condition-19	Test of Difference**
	(N = 38,327)	1143 (3%)	37,184 (97%)	
Males n, (%)	15,202 (40.0%)	412 (36.1%)	14,790 (39.8%)	
Females n, (%)	23,116 (60.0%)	730 (63.9%)	22,386 (60.2%)	p = 0.012
Age, years (mean, s.d.)	52.6 (18.3)	53.5(16.1)	52.6(18.3)	p = 0.006
BMI (mean, s.d.)	30.3 (7.1)	31.0 (7.4)	30.2 (7.1)	p < 0.001
Race				
White	26,237 (68.5%)	843 (73.8%)	25,394(68.3%)	
Black	1,234 (3.2%)	26 (2.3%)	1,208 (3.2%)	
Unknown	9,334 (24.4%)	234 (20.5%)	9,100 (24.5%)	
Other	1,522 (4.0%)	40 (3.5%)	1,482 (4.0%)	p = 0.001
Ethnicity				
Hispanic	2,519 (6.6%)	80 (7.0%)	2,439 (6.6%)	
Not Hispanic	22,100 (57.7%)	696 (60.9%)	21,404 (57.6%)	
Unknown	13,708 (35.8%)	367 (32.1%)	13,341 (35.9%)	p = 0.032
Ever Smoker Y/N (n = 14,591)	14,591 (38.1%)	437 (38.2%)	14,154 (38.1%)	p = 0.908
Systolic BP (mean, s.d.) n = 35,100	123.7 (15)	123.6 (15.1)	123.7 (15.4)	p = 0.968
Diastolic BP (mean, s.d.) n = 35,100	75.8 (8)	75.9 (8.6)	75.8 (8.9)	p = 0.410
Utilization**Ɨ** (mean encounters/month, s.d.)	2.4 (2.5)	3.3 (2.6)	2.3 (2.4)	<0.001

Abbreviations: s.d., standard deviation, BMI, body mass index (kg/m^2^). *Post COVID condition-19 patients had a diagnosis code of Post COVID condition ICD10: U09.9. ** p-value testing H0: no difference between Post COVID condition-19 and not Post COVID condition-19 patients. Chi-square test (categorical data), Wilcoxon (age, utilization-continuous data) and t-tests for BP (systolic and diastolic) data. ƗUtilization was counted as one encounter per day after first COVID Diagnosis or if that was not available, after first Post COVID condition Diagnosis. These represent the mean number of medical encounter-days per month of follow-up time post covid. Partial months of follow-up were set to one month.

### Post COVID condition symptoms recorded more than 30 days past COVID-19 or post COVID condition diagnosis

Fatigue, respiratory symptoms (shortness of breath, dyspnea), heart palpitations, disturbances of taste and smell, and postural orthostatic tachycardia syndrome (POTS) were all significantly more likely to be recorded in patients with Post COVID condition, however their prevalences were quite low. (Table 2) We found that these patterns persisted in patients under the age of 65 and were not observed in those 65 or older.

**Table 2:** Post COVID Condition Symptoms Overall and Stratified by age 65.

Variables	Total COVID-19 Patients		All Participants			Under 65 Years				>= 65 Years	
		Yes Post COVID Condition	No Post COVID Condition	*Test of Difference		Yes Post COVID Condition	No Post COVID Condition	*Test of Difference		Yes Post COVID Condition	No Post COVID Condition	*Test of Difference
Post COVID Condition Symptoms**, n (%)	n = 38,327	n = 1143	N = 37,184		N = 28,573	n = 871	n = 27,702		N = 9754	n = 272	n = 9,482	
Fatigue/Exhaustion	1654 (4%)	67 (6%)	1587 (4%)	p < 0.009	1128 (4%)	56 (6%)	1072 (4%)	p < 0.001	526 (5%)	11 (4%)	515 (5%)	p = 0.318
Dizziness on standing (lightheadedness)	1508 (4%)	53 (5%)	1455 (4%)	p = 0.215	871 (3%)	31 (4%)	840 (3%)	p = 0.373	637 (6.5%)	22 (8%)	615 (6%)	p = 0.292
Respiratory symptoms/sob/dyspnea	1100 (3%)	54 (5%)	1046 (3%)	p < 0.001	516 (2%)	34 (4%)	482 (2%)	p < 0.001	584 (6%)	20 (7%)	564 (6%)	p = 0.336
Fast-beating or pounding heart (also known as heart palpitations)	1255 (3%)	50 (4%)	1205 (3%)	p = 0.030	997 (4%)	40 (5%)	957 (3%)	p = 0.072	258 (2.7%)	10 (4%)	248 (3%)	p = 0.282
Disturbances of taste and smell	92 (<0.2%)	19 (2%)	73 (0.2%)	p < 0.001	61 (0.2%)	19 (2%)	42 (0.2%)	p < 0.001	31 (0.3%)	0	31 (0.3%)	p = 0.345
POTS: Postural orthostatic tachycardia syndrome	36 (0.1%)	5 (0.4%)	31 (0.1%)	p < 0.001	35 (0.1%)	5 (0.6%)	30 (0.1%)	p < 0.001	1 (0.01%)	0	1 (0.01%)	p = 0.865

*Chi -Square for Post COVID Condition Symptoms; **Disturbances/changes of taste and smell (R43.8, R43.9, R43.0); Fatigue/Exhaustion (R53.8x); Respiratory symptoms/SOB/dyspnea (J96.xx, J80.xx, R06.02, R06.03, R06.00) recorded in EMR ≥ 30 days after COVID (or if missing, Post COVID Condition) diagnosis.

### Pre-infection comorbidities and any autoimmune disease

Initially, we examined the crude relationships between PCC and comorbidities known or suspected to be associated with increased risk of severe COVID-19 (using tests of difference), as we had in our previous studies [22,23]. (Table 3) High BMI (>30) was more likely to be observed in the PCC diagnosed patients overall, in those under 65 years of age, as well as those 65 years and older. Overall, chronic respiratory disease was significantly more likely to be observed among patients with a PCC diagnosis. Among those under 65, hypertension, diabetes mellitus, and chronic respiratory disease were all significantly increased among patients with a PCC diagnosis. (Table 3) Our sensitivity analysis confirmed that these relationships were not confounded by COVID vaccination status, as there was essentially no change in the magnitude or significance of our findings. (See Supporting Information- S2 Appendix-Description of Study Codes and Algorithms and Supplemental Tables-Table 5)

**Table 3:** Comorbidities and Post COVID Condition* Stratified by Age.

		All Participants			Under 65 Years			>= 65 Years			
		N = 38,327			n = 28,573			n = 9,754			
	Total COVID	Yes Post COVID condition	No Post COVID condition	Test of Difference	Yes Post COVID condition	No Post COVID condition	**Test of Difference	Yes Post COVID condition	No Post COVID condition	**Test of Difference	
Comorbidities	N = 38327	n = 1143	n = 37184		n = 871	n = 27,702		n = 272	n = 9,482		
BMI High (>30 kg/m^2^)	15,768 (44.9%)	557 (50.3%)	15,211 (44.7%)	p < 0.001	435 (51.7%)	11,794 (46.5%)	p = 0.003	122 (45.7%)	3417 (39.7%)		p = 0.048
Hypertension	10,633 (27.7%)	330 (28.9%)	10,303 (27.7%)	p = 0.387	169 (19.4%)	4,545 (16.4%)	p = 0.019	161 (59.2%)	5758 (60.7%)		p = 0.610
Chronic Resp. Disease	9,513 (24.8%)	400 (35.0%)	9,113 (24.5%)	p < 0.001	276 (31.7%)	6,177 (22.3%)	p < 0.001	124 (45.6%)	2936 (31.0%)		p < 0.001
Diabetes Mellitus	4,670 (12.2%)	150 (13.1%)	4,520 (12.2%)	p = 0.325	83 (9.5%)	2,082 (7.5%)	p = 0.027	67 (24.6%)	2438 (25.7%)		p = 0.689
Chronic Kidney Disease	3,171 (8.3%)	79 (6.9%)	3,092 (8.3%)	p = 0.090	17 (1.9%)	492 (1.8%)	p = 0.699	62 (22.8%)	2600 (27.4%)		P = 0.091
Cancer	2,641 (6.9%)	62 (5.4%)	2,579 (6.9%)	p = 0.047	23 (2.6%)	909 (3.3%)	p = 0.295	39 (14.3%)	1670 (17.6%)		P = 0.161
Arterial Disease	2,388 (6.2%)	75 (6.6%)	2,313 (6.2%)	p = 0.638	22 (2.5%)	511 (1.8%)	p = 0.143	53 (19.5%)	1802 (19.0%)		p = 0.842
Congestive Heart Failure	1,332 (3.5%)	32 (2.8%)	1,300 (3.5%)	p = 0.205	12 (1.4%)	279 (1.0%)	p = 0.283	20 (7.4%)	1021 (10.8%)		p = 0.072
Polycystic Ovary Syndrome^Ɨ^	476 (1.2%)	17 (1.5%)	459 (1.2%)	p = 0.447	17 (1.9%)	455 (1.6%)	p = 0.481	4 (0.04%)	0 (0.0%)		p = 0.735
Immunosuppressed	356 (0.9%)	14 (1.2%)	342 (0.9%)	p = 0.290	10 (1.1%)	241 (0.9%)	p = 0.386	4 (1.5%)	101 (1.1%)		P = 0.523
Chronic Liver Disease	338 (0.9%)	10 (0.9%)	328 (0.9%)	p = 0.979	6 (0.7%)	173 (0.6%)	p = 0.813	4 (1.5%)	155 (1.6%)		P = 0.833

*Post COVID condition patients had a diagnosis code of Post COVID condition ICD10: U09.9. ** p-value testing H0: no difference between Post COVID condition and not Post COVID condition patients. Chi-square test (categorical data). Ɨ Polycystic Ovary Syndrome in females.

**Table 5:** Autoimmune Comorbidities and Post COVID condition* at two time periods: Pre-infection and post-infection.

	All Participants			
	N = 38,327			
	Adj OR** (95% CI)			
	Post COVID condition*			
	Total (N = 1143)			
Autoimmune Comorbidities	N (%)	*Pre-Infection*	N (%)	*Post-Infection*
*Any Autoimmune Disease Ɨ*	6757 (18%)	1.14 (0.98, 1.33)	*708 (1.9%)*	*1.57 (1.10, 2.24)*
	**n**	***Pre-Infection***	***n***	***Post-Infection***
Hashimoto thyroiditis	3380	1.03 (0.84, 1.27)	189	1.76 (0.93, 3.34)
Psoriasis	1104	1.41 (1.04, 1.91)	103	1.31 (0.48, 3.58)
Inflammatory Bowel Disease	856	1.32 (0.93-1.87)	125	1.05 (0.39, 2.85)
Graves’ Disease	433	0.89 (0.50, 1.58)	40	1.66 (0.40, 6.89)
Rheumatoid Arthritis	339	1.64 (1.00, 2.69)	32	3.18 (0.99, 10.46)
Sjögren’s Syndrome	389	1.38 (0.84, 2.26)	70	4.05 (1.94, 8.49)
Addison Disease	308	0.72 (0.34, 1.52)	69	0.46 (0.06, 3.32)
Chronic Fatigue Syndrome	173	1.14 (0.50, 2.58)	40	1.72 (0.41, 7.12)

*Post COVID condition – defined as ICD10: U09.9. **Adj OR = Adjusted odds ratio controlled for age and gender; Ɨ Any Autoimmune disease includes any of the following diagnoses prior to or post SARS-CoV-2 infection or if that was missing, Post COVID condition diagnosis: Addison disease, celiac disease, Graves’ Disease, Hashimoto thyroiditis, Inflammatory Bowel Disease (Crohn diagnosis, ulcerative colitis), Multiple Sclerosis, Myasthenia Gravis, Pernicious Anemia, Reactive Arthritis, Rheumatoid Arthritis, Sjögren’s syndrome, Systemic lupus erythematosus (lupus), Psoriasis, Lyme Disease, Chronic Fatigue Syndrome, Immunodeficiency following Epstein Barr. Small numbers of some auto-immune comorbidities (e.g., multiple sclerosis, systemic lupus erythematosus, Lyme disease, etc.) made it impossible to examine these diseases at the individual level.

We then examined these relationships using logistic regression models both crudely and while controlling for age and gender. Adjusted models demonstrated that overall, high BMI (>30): (OR= 1.25, 95% CI: 1.11, 1.41), chronic respiratory disease: (OR=1.64, 95% CI: 1.45, 1.86), and any post-infection autoimmune disease: (OR=1.57, 95% CI: 1.10, 2.24) were more likely to be observed in patients with a PCC diagnosis after controlling for age and gender. Pre-infection autoimmune diseases were marginally more likely to be observed in PCC diagnosed patients: (OR= 1.14, 95% CI: 0.98, 1.33) (Table 4).

**Table 4:** Logistic Regression Analyses of the association between Post COVID condition diagnosis* and pre-existing comorbidities.

	All Participants	
	N = 38,327	
	OR** (95% CI)	Adj OR*** (95% CI)
Comorbidities	Post COVID condition N = 1143	Post COVID condition* N = 1143
Hypertension	1.06 (0.93, 1.21)	1.02 (0.87, 1.18)
Diabetes Mellitus	1.09 (0.92, 1.30)	1.06 (0.88, 1.27)
BMI High^Ɨ^ (>30 kg/m^2^)	1.25 (1.11, 1.41)	1.25 (1.11, 1.41)
Chronic Resp. Disease	1.66 (1.47, 1.88)	1.64 (1.45, 1.86)
Arterial Disease	1.06 (0.83, 1.34)	1.02 (0.79, 1.32)
Congestive Heart Failure	0.79 (0.56, 1.13)	0.73 (0.51, 1.05)
Immunosuppressed	1.34 (0.78, 2.29)	1.33 (0.78, 2.28)
Chronic Renal Disease	0.82 (0.65, 1.03)	0.71 (0.55, 0.92)
Cancer	0.77 (0.59, 0.997)	0.72 (0.55, 0.93)
Chronic Liver Disease	0.99 (0.53, 1.87)	0.98 (0.52, 1.85)
Polycystic Ovary Syndrome ^Ɨ^	1.21 (0.74, 1.97)	1.26 (0.77, 2.06)
Pre-Infection Any Autoimmune Disease ^Ɨ Ɨ^	1.20 (1.04, 1.39)	1.14 (0.98, 1.330)
Post-Infection Any Autoimmune Disease ^Ɨ Ɨ^	1.61 (1.13, 2.29)	1.57 (1.10, 2.24)

*Post COVID condition diagnosis – defined as ICD10: U09.9. **OR = odds ratio, ***Adj OR = Adjusted odds ratio controlled for age and gender; ƗPolycystic Ovary Syndrome in females. ƗƗAny Autoimmune disease includes any of the following diagnoses prior to or post SARS-CoV-2 infection or if that was missing, Post COVID condition diagnosis: Addison disease, celiac disease, Graves’ disease, Hashimoto thyroiditis, inflammatory bowel disease (Crohn’s diagnosis, ulcerative colitis), multiple sclerosis, myasthenia gravis, pernicious anemia, reactive arthritis, rheumatoid arthritis, Sjögren’s syndrome, systemic lupus erythematosus (lupus), psoriasis, Lyme disease, chronic fatigue syndrome, immunodeficiency following Epstein Barr.

### Pre-infection and post-infection autoimmune diseases

Further exploration of the more prevalent autoimmune diseases using Logistic Regression models controlling for age and gender demonstrated that pre-infection, psoriasis and rheumatoid arthritis were more likely to be observed in the patients diagnosed with PCC. (Table 5) Post-infection diagnosis of Sjögren’s syndrome was significantly more likely and rheumatoid arthritis most likely to be found among patients with a PCC diagnosis after controlling for age and gender. Unfortunately, our power was limited and were unable to more fully explore each autoimmune disease included in the composite variable of “Any Autoimmune Disease” in these two time periods.

### Healthcare utilization

We observed significantly more medical-encounter days per month among patients diagnosed with PCC. (Table 6) This relationship was consistent when we examined it overall (2.33 vs 3.26, p < 0.0001), stratified by age 65 (<65: 1.90 vs 2.93, p < 0.001; ≥ 65 3.61 vs 4.31, p < 0.001) and gender (females: 2.48 vs 3.49, p < 0.001; males 2.11 vs 2.84, p < 0.001). In each stratum, we observed approximately one more day of utilization per month among COVID patients who were diagnosed with PCC.

**Table 6:** Healthcare Utilization*in those with and without Post COVID condition** Overall and Stratified by Age 65 and Gender.

	Post COVID condition Yes		Post COVID condition No		
Encounters per month	Mean	95% CI	Mean	95% CI	p-value
Overall	3.26	3.11, 3.41	2.33	2.31, 2.36	< 0.001
< 65 years	2.93	2.78, 3.09	1.90	1.88, 1.92	< 0.001
≥ 65 years	4.31	3.94, 4.68	3.61	3.55, 4.68	< 0.001
Females	3.49	3.30, 3.68	2.48	2.45, 2.51	< 0.001
Males	2.84	2.59, 3.09	2.11	2.07, 2.15	< 0.001

*Utilization was counted one encounter per day after first COVID-19 diagnosis or if that was not available, after first Post COVID condition diagnosis. These represent the mean number of medical encounter-days per month of follow-up time post covid. Partial months of follow-up were set to one month. ** Post COVID condition patients had a diagnosis code of Long COVID ICD10: U09.9

## Discussion

We have conducted two previous investigations of COVID-19 patients from a group medical practice located in central Massachusetts. In our first publication, we examined the relationship between anti-hypertensive medication use and risk of severe COVID-19 [22]. And in our second paper, we examined the association between markers of metabolic dysfunction and polycystic ovary syndrome and risk of severe COVID-19 in patients under 65 years [23]. In this paper we have expanded our study period and study size to explore PCC in this community-based population of patients who had COVID-19. We have examined the associations between PCC and demographics and comorbidities with a new focus on autoimmune diseases in two different timeframes: pre- and post-SARS-CoV-2 infection. We also examined medical utilization in relation to PCC diagnosis, overall and stratified by age and gender.

Our findings are consistent with others who demonstrate increased risk of PCC diagnosis among women and those with high BMI [26–28]. Our population is insured and largely white and non-Hispanic, benefiting from access to medical care, so it is not surprising we see more PCC diagnosis among non-Hispanic whites. Of the comorbidities we examined that have often been reported to be associated with severe COVID-19 (hospitalization or death), only high BMI (>30) and chronic respiratory disease were more likely to be observed in patients with a diagnosis of PCC in our population [27,29]. Deterioration in chronic obstructive pulmonary disease (COPD) manifested by worsening dyspnea and increase of severe exacerbations has been demonstrated in COPD patients after SARS-CoV-2 [30].

Our exploration of autoimmune diseases both pre- and post-SARS-CoV-2 infection adds to the growing body of evidence showing the complex relationships between this virus and autoimmune disorders. Women are much more likely to develop both autoimmune diseases and PCC, and it is not clear whether hormones, genetics or both play a role in these etiologies. Although we know there are genetic and environmental (e.g., viruses, diet, bacteria, drugs) causes of autoimmune diseases, the underlying mechanisms remain poorly understood. Potential mechanisms include generation of auto antibodies [31], molecular mimicry by viral proteins (viral sharing of amino acid sequences with self-antigens), epitope spreading secondary to release of self-antigens (continued autoimmune response that expose and present concealed self-antigens), and bystander activation of normally sequestered self-reactive T-cells and viral reservoirs via inflammatory mediators [32,33].

Viral persistence which occurs in some COVID-19 patients [34–36] is another potential mechanism enhancing activation of auto-reactive T- or B- cells. The inflammatory process plays a significant role in severe COVID-19 and in the development and prognosis of autoimmune diseases. This indicates that inflammation likely plays an important role in the relationship between SARS-CoV-2, PCC and the exacerbation of existing and onset of new autoimmune disorders. The intricacies of these relationships need further exploration.

We were able to demonstrate that overall, COVID-19 patients with any of the post-infection autoimmune disorders we examined were more likely to be among patients with a PCC diagnosis. There was a strong suggestion that patients with autoimmune diagnoses prior to infection with SARS-CoV-2 were also more likely to be diagnosed with PCC. Although our power was somewhat limited, we were able to explore a subset of specific autoimmune diseases (Hashimoto thyroiditis, psoriasis, inflammatory bowel disease, Graves’ disease, rheumatoid arthritis, Sjögren’s syndrome, Addison Disease and chronic fatigue syndrome). Among the autoimmune diseases investigated, pre-infection psoriasis was associated with a PCC diagnosis. Song and colleagues argue that patients with psoriasis are at increased risk for more severe COVID-19 outcomes which could partially explain this finding [37].

Post infection, Sjögren’s syndrome showed a strong association with PCC with a 4-fold increase in risk but our numbers were modest so there is a wide confidence interval around that estimate. Brito-Zeron followed 132 patients with Sjögren’s syndrome and found that 57% of these patients remained symptomatic with post COVID-19 syndrome after a mean follow-up of 5 months. Chang reported a slightly smaller magnitude of effect for newly reported Sjögren’s syndrome (HR 2.62; 95% CI: 2.29–3.00) after SARS-CoV-2 and Tesch roughly a 40% increase in risk (IRR 1.44; 95% CI: 1.27–1.63) [38–40].

There are mixed findings in the relationship between rheumatoid arthritis and COVID-19 manifestations, including PCC [16,41–43]. Interestingly, our findings suggest that rheumatoid arthritis seemed to be more prevalent among patients with a PCC diagnosis regardless of whether the diagnosis was before or after their SARS-CoV-2 infection. Prior to the COVID-19 pandemic, an ecologic study examining the relationship between various respiratory viruses and incident rheumatoid arthritis found a strong association with corona viruses [44]. Chang and colleagues report a three-fold risk of newly reported RA post SARS-CoV-2 infection among COVID-19 patients [39]. Wuller and colleagues recently demonstrated that severe acute COVID was most strongly associated with risk for new autoimmune disease as compared to those with less severe disease [16].

Patients suffering from autoimmune diseases shown to increase risk of severe COVID-19 or onset of PCC should be targeted for preventive care including vaccination [45] and possibly treatment with antiviral medications [46] in consultation with their medical team. Efforts to reduce the risk of severe COVID-19 in these patients seem especially important. Providers should be alert to the possible onset of autoimmune diseases in patients post SARS-CoV-2 infection.

Our findings regarding increased medical utilization among those with a PCC diagnosis have been confirmed by others. Koumpias and colleagues demonstrated that healthcare utilization was significantly higher among COVID-19 patients over the 6-month period after they were diagnosed when compared to the pre-diagnosis period [47]. Bowe and colleagues examined the sequelae of COVID-19 at 2 years post-acute SARS-CoV-2 infection and report that while risk of many sequelae declined 2 years after the infection, still there was a “substantial cumulative burden of health loss due to PASC” [48].

PCC is associated with sizable increases in health care service utilization and therefore, medical costs [26,47,49,50]. As the SARS-CoV-2 virus mutates the rate of resulting incident PCC may be dropping. For example, the risk of PCC after Omicron (4.5%) was about half of that estimated for Delta (10.8%) [51]. However these variants have shown to be more contagious, and many more people contracted the Omicron variant than Delta so the sum total of PCC patients remains substantial [52]. Due to limitations in our dataset, we were unable to explore the impact of multiple variants. Nevertheless, our findings highlight the importance of planning proper resource allocation for patients suffering from PCC.

Strengths of our study include the prospectively collected EHR data from a well-studied Massachusetts community-based population. We conducted chart reviews to ensure those patients with a PCC diagnosis had documentation of a prior SARS-CoV-2 infection which helped to validate our case definition. Many of these patients who were missing acute COVID-19 diagnoses in the EHR, likely used over the counter rapid antigen tests or sought testing or care outside the group medical practice. We used a provider diagnosis of PCC to identify cases, however diagnosis of PCC can be subjective as symptoms can persist, resolve and re-emerge [6]. Our prevalence of PCC diagnosis was relatively low compared to other reports [2]. In addition, we did not have access to viral load or clearance data so could not precisely determine post-COVID condition. It is also possible that in some cases, post-intensive care syndrome may have been misclassified as post COVID condition. We used the optimal definition of PCC given the EHR data we had but also understand that we have likely missed the less severe cases of PCC (and PCC symptoms), who were not troubled enough to seek medical care and may not have properly classified all post-COVID condition cases.

There are other potential limitations that must be considered, including the possibility that autoimmune diagnoses are likely underestimated in EHR data [53]. Patients who seek routine medical care (have higher utilization), including those with autoimmune diseases may be more likely to be diagnosed with PCC than those not seeking regular medical care. And a diagnosis of PCC may also increase medical utilization and thus the chance of an autoimmune disease diagnosis. So, although these results are interesting and provide some potential useful information, our findings need to be reproduced in prospective cohort studies specifically designed to assess the link between COVID-19/long COVID and autoimmune disorders.

The timeframe of COVID-19 symptom persistence indicating a PCC diagnosis has also changed. Initially it was symptoms lasting 4 weeks or more but subsequent studies use even longer timeframes, including symptoms lasting 3 months or longer [54,55]. Although possible, we doubt that these changes in definition will alter the direction or strength of our findings. It is also important to recognize that PCC could represent a constellation of systemic autoimmune disorders, and the preclinical phase of autoimmune disorders can last longer than a decade so the definite conclusion about whether COVID-19 and/or PCC lead to autoimmune disorders will require many more years of investigation [56]. It is important to note that our insured and largely white, non-Hispanic cohort may not be representative of the general US population so generalization of our results should be made with caution.

## Conclusions

Our findings suggest that potential risk factors for PCC include high BMI (>30 kg/m^2^), female gender, and prior history of chronic pulmonary disease. Further there is a complex relationship between autoimmune diseases and SARS-CoV-2 that warrants additional investigation in a larger population. Our findings support the emerging evidence that pre-existing autoimmune diseases may increase the risk of PCC diagnosis and that conversely SARS-CoV-2 may increase the risk of new onset autoimmune diseases in some patients. Future prospective and longitudinal investigations of both scenarios are essential to expand our understanding. The etiologic role of the inflammatory process should be more closely examined, and providers should more carefully monitor patients with autoimmune disorders and high BMI post SARS-CoV-2 infection. And finally, given the complexity of COVID-19 sequelae, healthcare utilization is higher among patients with a PCC diagnosis and appropriate healthcare resource planning is necessary.

## Supporting information

S1 Appendix RECORD checklist. (DOCX)

S2 Appendix Description of study codes and algorithms and supplemental tables. (DOCX)

## List of abbreviations

BMI: Body Mass Index
BP: Blood pressure
95% CI: 95% Confidence Interval
COVID-19: Coronavirus Disease 2019
EHR: Electronic Health Record
HT: Hypertension
OR: Odds ratio; PASC-Post-acute sequelae of COVID-19
PCC: Post COVID condition
PCOS: Polycystic Ovary Syndrome
PCR: Polymerase chain reaction
RT-PCR: Reverse transcription polymerase chain reaction
SARS-CoV-2: Severe acute respiratory syndrome coronavirus 2
