# Supplementary material for: Autoimmune diseases as pre-existing conditions and sequelae of post COVID-19 condition in a Massachusetts community based observational study of COVID-19 patients
Source: PLoS One. 2025 Dec 3;20(12):e0337848. doi: 10.1371/journal.pone.0337848 (PMC12674521; doi:10.1371/journal.pone.0337848)
Supplement: S2 Appendix — (DOCX) [file pone.0337848.s002.docx]

**S2 Appendix: Description of Study Codes and Algorithms and Supplemental Tables**

**Identification of COVID Patients for Study Sample:** CPT Codes for SARS-COV-2 reverse transcription polymerase chain reaction, RT-PCR (87635.xx and 87798.189, 87636.01), Rapid Antigen tests (87426.xx) with positive result and ICD-10 Codes were used by the RMG data analyst to extract patients with COVID-19 (U07.1) and Long COVID-19 (U09.9) diagnoses. (Tables 1 and 2)

| **Table 1. Procedure Codes for Identifying Patients with COVID-19** | |
| --- | --- |
| **Procedure Code** | **Procedure Name** |
| 87635.xx | SARS CORONAVIRUS W/COV2 RNA, QL RT-PCR |
| 87798.189 | SARS-COV-2 RNA, QUALITATIVE REAL-TIME RT-PCR |
| 87636.01 | COVID-19 ANTIGEN RAPID TEST (STATION) |
| 87426.xx | COVID-19 ANTIGEN HOME TEST |

| **Table 2. ICD-10 Code for Identifying Patients with COVID-19 and Long COVID** | |
| --- | --- |
| **ICD-10 Code** | **Description** |
| U07.1 | COVID-19 |
| U09.9 | Long COVID-19 (PASC) |

**Time Period:** Data Pull included records from: 1/1/2020 to 06/25/2023.

**Outcome:**

All identified COVID-19 patients were divided into two groups comparing those with a Long COVID diagnosis to those without a Long COVID diagnosis.

**Confounders and Effect Modification:**

**Age** was calculated based on 1/1/2020 and will require pts included are >= 18 at that timepoint. This ensures we don’t include records from “Minors” at any point. Only those age >=18 will be included for extraction and analysis.

We evaluated the confounding effects of age (continuous) and gender (categorical) in our logistic regression models. Effect Modification was not evaluated in our logistic regression modeling.

**Diagnosis Codes:** ICD-10 codes associated with encounter data (prior to first COVID-19 diagnosis) were used to categorize presence or absence of Long COVID symptoms (Table 3) and comorbidities (Table 4)

Demographic variables including age, gender, race, ethnicity, BMI, and smoking status were also extracted from the Electronic Health Record. To improve accuracy, we extracted the last reported value prior to the COVID-19 diagnosis for smoking status and BMI.

**Utilization:** Utilization was counted one encounter per day after first COVID Dx or if that was not available, after first Long COVID Diagnosis. These represent the mean number of medical encounter-days per month of follow-up time post covid. Partial months of follow-up were set to one month.

| **Table 3. Long COVID Symptoms and ICD 10 Codes** | |
| --- | --- |
| **Long COVID Symptoms*** | **ICD 10 Codes** |
| Disturbances of taste and smell | R43.8, R43.9, R43.0 |
| Fatigue /Exhaustion | R53.8x |
| Respiratory symptoms/sob/dyspnea | J96.xx, J80.xx, R06.02, R06.03, R06.00 |
| Fast-beating or pounding heart (also known as heart palpitations) | R00.2 |
| Dizziness on standing (lightheadedness) | R42 |
| POTS: Postural orthostatic tachycardia syndrome | G90.A |

*Symptoms recorded in EMR ≥ 30 days after COVID (or if missing, Long COVID) diagnosis.

| **Table 4. Comorbidities and Associated ICD-10 Codes.** | |
| --- | --- |
| **Comorbidity** | **ICD-10 Code** |
| Hypertensive diseases | I-10 -I16 |
| Coronary artery disease | I20-I25 |
| Congestive heart failure | I50.x |
| Diabetes | E8-E13 |
| Chronic Respiratory Disease  Asthma  Chronic Obstructive Pulmonary Disease  Emphysema  Obstructive Sleep Apnea | J45.x  J44.x  J43.x  G47.3x |
| Immunosuppression  HIV  History of solid organ transplant | B20.x, Z20.xx  Z94.x |
| Kidney Disease  Chronic  End stage | N18.x  N18.6x |
| Chronic Liver Disease  Cirrhosis  Chronic Hep B  Chronic Hep C | K74.6x  B18.1x  B18.2x |
| Cancer | Cxx.x |
| Polycystic Ovary Disease | E28.2 |
| Obesity | 30 > BMI ≥ 30 |
| **Autoimmune Diseases** |  |
| Addison Disease | E27.x |
| Celiac Disease | K90.0 |
| Graves’ Disease | E05.x |
| Hashimoto thyroiditis | E06.3, E02, E03.x |
| Inflammatory Bowel Disease (Crohn dx, ulcerative colitis) | K50-K52 |
| Multiple Sclerosis | G35 |
| Myasthenia Gravis | G70.x |
| Pernicious Anemia | D51.0 |
| Reactive Arthritis | M02.* |
| Rheumatoid Arthritis | M06.9; M05.x |
| Sjögren syndrome | M35.00-.09 |
| Systemic lupus erythematosus (lupus) | M32.x |
| Psoriasis | L40.x |
| Lyme Disease | A69.2x ( A69.20, A69.21, A69.22, A69.23, A69.29) |
| Chronic Fatigue Syndrome | R53.82 |
| Immuno-deficiency following Epstein Barr | D82.3 |

| **Table 5. Autoimmune Comorbidities and Long COVID* at two time periods: Pre-infection and post-infection overall and among those with COVID vaccination**. | | | | |
| --- | --- | --- | --- | --- |
| **All Participants**  **N=38,327**  **Adj OR** (95% CI)**  **Long COVID***  **Total (N= 1143 )** | | | | |
| **Autoimmune Comorbidities** | N (%) | ***Pre-Infection*** | N (%) | ***Post-Infection*** |
| Any Autoimmune DiseaseƗ | 6757(18%) | 1.14 (0.98, 1.33), p=0.083 | *708 (1.9%)* | *1.57 (1.10, 2.24), p=0.013* |
| Only Vaxed Any Autoimmune DiseaseƗƗ | 5704 (19%) | 1.14 (0.97, 1.35), p=0.107 | *604 (2%)* | *1.62 (1.12, 2.36), p=0.011* |
| *Long COVID-19 – defined as ICD10: U09.9. **Adj OR = Adjusted odds ratio controlled for age and gender; Ɨ Any Autoimmune disease includes any of the following diagnoses prior to or post SARS-CoV-2 infection or if that was missing, Long COVID diagnosis: Addison disease, celiac disease, Graves’ Disease, Hashimoto thyroiditis, Inflammatory Bowel Disease (Crohn diagnosis, ulcerative colitis), Multiple Sclerosis, Myasthenia Gravis, Pernicious Anemia, Reactive Arthritis, Rheumatoid Arthritis, Sjögren’s syndrome, Systemic lupus erythematosus (lupus), Psoriasis, Lyme Disease, Chronic Fatigue Syndrome, Immunodeficiency following Epstein Barr. Ɨ Ɨ Only Vaxed Any Autoimmune disease is as above but restricted to include only patients with at least one COVID-19 vaccination (n=30,690). Total LC diagnosed patients with at least one COVID-19 vaccination n=945. | | | | |
